# Ophthalmologist and Optometrist Comanagement Model for Glaucoma: Outcomes from a Single Tertiary Care Health Center

**DOI:** 10.1016/j.ogla.2026.02.002

**Published:** 2026-02-26

**Authors:** Evan M. Chen, Samantha Chan, Kelly J. Chen, Ashika Kuchhangi, Matthew D. Huh, Jill Liang, Patrick M. Takla, Eva Pak, Jenny H. Lu, Aishwarya Vadivel, Dake Chen, Julius Oatts, Jing Shan, Yinxi Yu, Gui-Shuang Ying, Joshua D. Stein, Tin Aung, Taras Litvin, Ying Han

**Affiliations:** 1 Department of Ophthalmology, University of California, San Francisco, California.; 2 Division of Ophthalmology, Children’s Hospital of Philadelphia, Scheie Eye Institute, Perelman School of Medicine at the University of Pennsylvania, Philadelphia, Pennsylvania.; 3 Center for Preventive Ophthalmology and Biostatistics, Department of Ophthalmology, Scheie Eye Institute, Perelman School of Medicine, University of Pennsylvania, Philadelphia, Pennsylvania.; 4 Department of Ophthalmology and Visual Sciences, University of Michigan, Ann Arbor, Michigan.; 5 Department of Health Management and Policy, University of Michigan School of Public Health, Ann Arbor, Michigan.; 6 Singapore Eye Research Institute, Singapore National Eye Centre, Singapore, Singapore.; 7 Duke-NUS Medical School, National University of Singapore, Singapore, Singapore.; 8 The Francis I. Proctor Foundation for Research in Ophthalmology, San Francisco, California.; 9 Ophthalmology Section, Surgical Service, San Francisco Veterans Affairs Medical Center, San Francisco, California.

**Keywords:** Comanagement, Glaucoma, Optometry

## Abstract

**Purpose:**

The growing demand for eye care poses significant challenges to the effective management of patients with glaucoma. This study evaluated long-term clinical outcomes of patients with glaucoma comanaged by institutionally trained optometrists and glaucoma specialists at the University of California San Francisco.

**Design:**

Retrospective cohort study.

**Participants:**

Patients with glaucoma suspect or stable glaucoma diagnoses from 2016 to 2023.

**Methods:**

Patients were comanaged by glaucoma specialists and optometrists with postgraduate institutional glaucoma-specific training via either “alternate care” (alternating glaucoma specialist and optometrist visits) or “transfer care” (optometrist visits only) for up to 5 years of follow-up. Generalized linear models were used to assess long-term changes in clinical parameters.

**Main Outcome Measures:**

Changes in intraocular pressure (IOP), best-corrected visual acuity (BCVA), medications, Humphrey visual field (HVF), and retinal nerve fiber layer (RNFL) thickness.

**Results:**

The study included 391 patients (775 eyes), with 136 (34.8%) in alternate care and 255 (65.2%) in transfer care. The most common diagnoses were glaucoma suspect (51.2%) and primary open-angle glaucoma (POAG, 36.0%). No significant changes were observed in BCVA (*P* = 0.34), IOP (*P* = 0.18), medications (*P* = 0.11), or RNFL (*P* = 0.65) whereas the mean deviation of HVF improved (*P* = 0.003) over 5 years among both groups. Overall, 132 eyes (17.0%) required treatment escalation: 81 (10.5%) needed increased medications, 34 (4.4%) underwent glaucoma-related laser treatment, and 17 (2.2%) had incisional surgery. Among all eyes, 56 (14.3%) were rereferred to glaucoma specialists, with 43 (11.0%) returning to comanagement after evaluation or treatment. Alternate care patients saw both optometrists and glaucoma specialists at a median rate of once per year. Transfer care patients saw optometrists at a median rate of twice per year and none by glaucoma specialists. In multivariable analysis, escalation was associated with older age (odds ratio [OR], 1.30 per decade; 95% CI, 1.04–1.63; *P* = 0.02), POAG vs. suspect (OR, 2.99; 95% CI, 1.59–5.65; *P* < 0.001), and higher baseline IOP (OR, 1.18 per mmHg; 95% CI, 1.07–1.30; *P* = 0.01). Care pathway assignment was not significantly associated with treatment escalation.

**Conclusions:**

A well-defined and closely monitored comanagement model can maintain patient safety and glaucoma stability over 5 years with improved resource allocation and reduced utilization of glaucoma specialists.

The expanding and aging population in the United States presents a growing challenge for the management of patients with chronic eye disease. This creates the need to identify strategies to optimize eye care access, particularly for conditions requiring long-term specialized management such as glaucoma. Ensuring that glaucoma specialists are available to manage patients with complex, progressive disease requires an increase in the efficiency of eye care delivery.

The majority of patients with glaucoma have stable and mild disease^1–3^ and receive care from optometrists in real-world settings. Although optometrists without specialty-specific training have been shown to have agreement with ophthalmologists in terms of medical management decisions for mild glaucoma, concordance when interpreting visual field results and assessment of optic nerve damage, key components in glaucoma monitoring, was more limited.^4,5^ In contrast, studies conducted in the United Kingdom found that optometrists with glaucoma-specific training or formal accreditation made satisfactory decisions regarding the diagnosis, treatment, and follow-up intervals of patients with glaucoma.^6,7^ This was reported in practice at Moorfields Eye Hospital, where an integrated optometrist demonstrated good agreement with ophthalmologists with regard to optic disc assessment, management recommendations, and follow-up intervals.^8^ In Australia, the Centre for Eye Health identified approximately 188 patients suitable for management by optometrists and piloted a comanagement system, although outcome data from this initiative are not yet available.^9^

Although these emerging examples of glaucoma comanagement are promising, there remains a substantial gap in our understanding of the effectiveness and outcomes of comanagement models involving optometrists and ophthalmologists. To date, no US-based studies have assessed outcomes of glaucoma comanagement systems that could improve the efficiency of glaucoma care delivery by enabling glaucoma specialists to focus on patients with unstable or more severe glaucoma. Additionally, research has yet to assess the long-term outcomes of patients managed in these systems. Lastly, existing studies are limited by their small sample sizes and the inclusion of only a single optometrist, limiting the generalizability of findings. Therefore, the purpose of this study was to describe the longitudinal outcomes of patients comanaged by optometrists with institutional glaucoma-specific training and glaucoma specialists at a tertiary care center in the United States.

## Methods

This retrospective cohort study was approved by the University of California San Francisco (UCSF) Institutional Review Board and conducted in accordance with the Health Insurance Portability and Accountability Act. This study also adhered to the tenets of the Declaration of Helsinki. The requirement for informed consent was waived due to the retrospective nature of the study.

### Study Population

The electronic medical records of all patients aged 18 years or older who received outpatient care at the UCSF Department of Ophthalmology for glaucoma and glaucoma suspect status from January 2016 to November 2023 were reviewed. All patients participating in the comanagement system, as determined by review of clinical documentation from outpatient encounters, were included. Patients were required to have at least 1 follow-up appointment with an optometrist for glaucoma care after being designated for transfer or alternate care by a glaucoma specialist. The date on which the glaucoma specialist referred the patient to the comanagement system was deemed the baseline date. Patients were followed for up to 5 years after the baseline date. Follow-up intervals included were 1 year ± 3 months, 1.5 years ± 3 months, 2 years ± 6 months, 3 years ± 6 months, 4 years ± 6 months, and 5 years ± 6 months. Clinical visit data closest to each follow-up interval and within the specified window were recorded. Visits for refraction, postoperative care, or other nonglaucomatous conditions were excluded.

### Comanagement System

Beginning in January 2016, the UCSF Department of Ophthalmology implemented a protocol for a comanagement program of coordinated care between institutionally trained optometrists and glaucoma specialists defined as glaucoma fellowship-trained ophthalmologists (Fig 1). Participating optometrists completed a structured curriculum comprising didactic instruction, a minimum 3-week shadowing period, and a supervised trial phase with direct oversight by glaucoma specialists. Competency was evaluated through direct observation and review of clinical documentation before independent practice. Interobserver agreement was not formally assessed in this study, as this has been previously described in the literature.^4–7^ In total, 4 glaucoma specialists and 3 optometrists participated in the comanagement system.

Within the comanagement system, glaucoma specialists identified patients under their clinical care with glaucoma suspect status including ocular hypertension, or stable glaucoma. At the discretion of glaucoma specialists, patients were offered the opportunity to participate in a comanagement system comprising 2 pathways: (1) alternate visits with their current glaucoma specialist and a UCSF glaucoma-trained optometrist (alternate care) or (2) sequential visits with a UCSF glaucoma-trained optometrist alone, unless signs of disease progression required treatment escalation (transfer care). The glaucoma specialist determined which pathway a patient would enter. Stable glaucoma status was determined by glaucoma specialists and required no current or recent treatment escalation as well as clinical metrics without concern for disease progression.

Patients receiving care under optometrists, regardless of the comanagement type, were rereferred from optometrists back to glaucoma specialists when deemed to be at risk of progression based on elevated intraocular pressure (IOP) beyond target IOP previously determined by the glaucoma specialist in at least 2 consecutive visits, or deterioration in reliable Humphrey visual field (HVF) testing, OCT retinal nerve fiber layer (RNFL) imaging or new optic disc hemorrhage. The need for rereferrals was determined by optometrists. All treatment escalations were performed or approved by glaucoma specialists. Glaucoma specialists could return patients to the comanagement system after escalation of treatment or affirmation of stable disease. Referral for cataract surgery without a concurrent glaucoma procedure was not considered a transfer of care. Such patients resumed comanagement after postoperative recovery unless the disease course or sustained elevation in postoperative IOP warranted ongoing glaucoma specialist oversight. Patient movement from alternate to transfer care occurred at the discretion of the glaucoma specialist when patients achieved a level of disease stability consistent with transfer criteria. Appointments with optometrists were assigned based on availability; no optometrist was paired exclusively with any specific glaucoma specialist. After entry into the comanagement system, patients were followed by the same optometrist over time.

### Clinical Data

All patients were required to have a diagnosis of ocular hypertension, glaucoma suspect, or other type of glaucoma. Glaucoma diagnoses were further subdivided into primary open-angle glaucoma (POAG), mixed mechanism glaucoma, narrow-angle glaucoma (primary angle closure, primary angle closure glaucoma or plateau iris), pseudoexfoliation glaucoma, normal tension glaucoma, pigmentary glaucoma, uveitic glaucoma, traumatic glaucoma, and other glaucoma types. Other baseline clinical data were gathered, including ocular history, lens status (phakic, pseudophakic, and aphakic), history of laser or surgical glaucoma procedures, best-corrected visual acuity (BCVA), IOP, and number of glaucoma medications in each eye. Demographic factors included patient age, self-reported gender, and race. For HVF testing, mean deviation, pattern standard deviation, visual field index, and type of visual field test were included. All visual field testing was performed with the Humphrey Field Analyzer (Carl Zeiss Meditec). For OCT testing, the average, superior, and inferior RNFL thickness were collected. OCT testing was performed using the iVue (Optovue, Inc) and Cirrus (Carl Zeiss Meditec) platforms. Due to the limited number of patients with OCT measurements from the Cirrus platform (14 eyes, 3.6%), data from this platform were excluded from longitudinal analysis.

For clinical imaging, including HVF testing or OCT, any testing occurring at or within 3 months before the baseline visit date was recorded as baseline imaging data. Subsequent imaging data requirements followed the same time window criteria as clinical data; only those tests performed within the specified time period of each follow-up date were included. If multiple imaging tests were performed, the tests performed on the closest date to the follow-up date were included.

### Outcomes

The primary outcomes of this study were longitudinal changes in BCVA, IOP, mean deviation of HVF, and mean RNFL thickness. Humphrey visual field progression was defined by a decline in mean deviation >1 dB/year among patients with at least 3 examinations, a definition that has been adopted in other studies.^10,11^ Although there is a lack of consensus on what rate of RNFL thinning indicates progression, we utilized a conservative progression threshold of >1 μm/year decline for patients with at least 3 examinations in the iVue system.^12^ The secondary outcome of this study was treatment escalation, defined as an increase in the number of glaucoma medications, the need for a new glaucoma-related laser procedure, or new glaucoma surgery. We assessed the frequency and timing of treatment escalation from optometrists to glaucoma specialists. Additionally, we studied the clinical risk factors associated with treatment escalation. Lastly, to examine care utilization patterns in a comanagement system, this study identified the median number of clinical visits with glaucoma specialists and optometrists after initiation of comanagement care.

### Statistical Analysis

R 3.6.0 (R Foundation for Statistical Computing) and SAS v9.4 (SAS Institute) were used for all statistical analyses and graphical representations. Descriptive statistics, including mean and standard deviations (SD), were used for continuous measures, and counts with percentages were used for categorical measures. Unpaired Student *t* tests were used for continuous measures and chi-square tests for categorical measures were utilized to compare the baseline characteristics of patients in the transfer and alternate care pathways. Continuous variables were evaluated for normality using the Shapiro–Wilk test; nonnormal variables were analyzed using nonparametric tests. Generalized linear models for longitudinal data were used to assess changes in clinical parameters over time within the same comanagement group. Univariable and multivariable logistic regression models were used to identify factors associated with treatment escalation (i.e., loss of disease stability) and to account for differences in baseline factors. In univariable analysis, age, gender, comanagement type, glaucoma diagnosis, phakic status, history of glaucoma laser procedures, BCVA, IOP, number of glaucoma medications, visual field, and OCT metrics were included. Factors with a *P* value ≤ 0.2 from the univariable analysis were included in the initial multivariable model, which underwent forward variable selection by retaining only factors with *P* < 0.05 in the final multivariable model. Analyses were conducted on a patient and eye level. For eye-level analysis, the intereye correlation was accounted for using generalized estimating equations.

## Results

This study included 391 patients: 136 (34.8%) in the alternate care group and 255 (65.2%) in the transfer care group. Baseline features of the study cohort are reported in Table 1 for patient-level characteristics and in Table 2 for eye-level characteristics. The mean age of the cohort was 71.0 years (SD 13.0 years), 206 patients were women (52.7%) and 143 were Asian (36.6%). Eyes in the alternate care group were, on average, receiving a greater number of glaucoma drops (*P* = 0.003), were more likely to be phakic (*P* = 0.007), and to have a history of a prior glaucoma laser procedure (*P* = 0.007). The majority of patients entered into the comanagement system in 2018 (22.5%) and 2019 (27.6%). The most common diagnoses were glaucoma suspect (51.2%) and POAG (36.0%). Regarding prior glaucoma treatment, 78.3% of eyes had no history of laser surgery, including laser peripheral iridotomy or selective laser trabeculoplasty, and 92.4% had no history of incisional glaucoma surgery or microinvasive glaucoma surgery. There were no significant differences in BCVA, IOP, HVF mean deviation, or average RNFL thickness in the alternate and transfer care groups (Table 2).

### Longitudinal Analysis of Clinical Parameters

Over 5 years, individual-level analyses demonstrated no significant change in logMAR BCVA (*P* = 0.14), IOP (*P* = 0.25), average RNFL thickness (*P* = 0.52), or HVF mean deviation (*P* = 0.37) from baseline at all time points. When delineating eyes by alternate or transfer care group assignment, there were also no significant mean changes from baseline in these metrics. Figure 2A, B depict longitudinal changes from baseline in BCVA, IOP, mean RNFL, and HVF mean deviation over time, plotted with 95% confidence interval (CI) error bars for the alternate and transfer care pathways, respectively. Lastly, there was no significant change in the mean number of glaucoma medications used in this cohort (0.7–0.6 from baseline to 5 years, *P* = 0.11). In this study, 9.5% of included eyes underwent cataract surgery during the follow-up period. As cataract surgery can improve BCVA, mean deviation, and IOP, we conducted a sensitivity analysis censoring these eyes at the time of surgery, which still demonstrated stability in all clinical parameters. We also conducted a separate sensitivity analysis excluding HVFs with fixation losses > 20% or false-positive rates > 15% and OCTs with signal strength <6, which showed no change in the direction or significance of results.

### Care Utilization

Of the 255 patients assigned to the transfer pathway, 214 (83.9%) remained in this pathway throughout the study and did not require escalation of management with a glaucoma specialist (Table 3). Among the 41 patients who required further management by a glaucoma specialist, 30 were ultimately rereferred back to optometrist care after evaluation and possible treatment escalation. Eleven patients were referred to a glaucoma specialist and remained under their care due to requiring ongoing management for worsening disease.

In total, 136 patients were assigned to the alternate care path, of which 87 (64.0%) remained during the duration of the study (Table 3). Thirty-four (25.0%) patients in the alternate pathway were later sent to the transfer pathway and continued with optometrist management. Fifteen patients (11.0%) in the alternate care pathway were rereferred to a glaucoma specialist for continued care.

When examining utilization of glaucoma specialists after entering the comanagement system, patients in the transfer care group had a median of 2 visits to an optometrist per year and none to a glaucoma specialist. In the alternate care group, patients had a median of 1 visit to an optometrist and 1 visit to a glaucoma specialist per year.

### Rereferral to Glaucoma Specialists

In total, 56 eyes of 56 patients from either the transfer or alternate care group were rereferred back to glaucoma specialists. This rereferral occurred due to IOP being above goal for 29 (51.8%), progressive thinning of the RNFL for 14 (25.0%), and worsening HVF deficits for 13 (23.2%). The median time from referral by the optometrist to being seen by the glaucoma specialist was 86 days (interquartile range [IQR], 63–106 days). The delay in follow-up―the median difference between the specified follow-up interval per the optometrist and the actual follow-up interval―was 5 days (IQR, 0–39 days). Eight patients were recommended to follow up with a glaucoma specialist at the next available appointment rather than at a specified interval. These patients were seen a median of 28 days (IQR, 14–57 days) after the referral was placed for rereferral to the glaucoma specialist. There were no significant differences in the IOP or BCVA on the date of optometrist rereferral and the actual date of glaucoma specialist follow up. Patients rereferred to glaucoma specialists did not differ statistically significantly in demographics or baseline characteristics from the overall study cohort.

Among those rereferred to glaucoma specialists, 20 (5.1%) received an additional IOP-lowering medication simultaneously with recommending escalation of care. For the remaining 36 (9.2%), 11 (2.8%) received selective laser trabeculoplasty (SLT), 6 (1.5%) received new topical drops, and 2 (0.5%) received cataract surgery combined with microinvasive glaucoma surgery. The remainder were deemed stable and did not require further treatment.

### Treatment Escalation

At baseline, the mean number of glaucoma medications among all eyes was 0.7 (SD 1.0). Eyes in the alternate care pathway had a slightly greater mean number of glaucoma medications (1.0, SD 1.0) than those in the transfer care pathway (0.6, SD 1.0) (*P* = 0.003). Eyes in the alternate care group also more commonly required an increase in the number of glaucoma medications compared with those transferred for care (13.2% vs. 8.9%, *P* = 0.17), although this difference was not statistically significant. The alternate care pathway also had a non-statistically significant greater proportion of eyes that required a new laser procedure (5.1% vs. 4.0%, *P* = 0.54) or glaucoma surgery (4.4% vs. 1.0%, *P* = 0.02) during the follow-up period compared with the transfer pathway (Table 3). The average time to treatment escalation for any type of therapy in either group was 628 days.

### Factors Associated with Treatment Escalation

Overall, 132 (17.0%) eyes met criteria for treatment escalation from either the transfer or alternate care group due to an increase in drop utilization, laser trabeculoplasty, or surgery. In univariable analysis (Table S4, available at www.ophthalmologyglaucoma.org) to identify factors associated with treatment escalation during the study period, older age (odds ratio [OR], 1.29 per 10 years increase; 95% CI, 1.07–1.56; *P* = 0.008), alternate care compared with transfer of care (OR 1.87; 95% CI, 1.11–3.16; *P* = 0.02), POAG compared with glaucoma suspect disease status (OR, 2.99; 95% CI, 1.59–5.65; *P* < 0.001), history of selective laser trabeculoplasty (OR, 2.48; 95% CI, 1.36–4.50; *P* = 0.003), higher baseline IOP (OR, 1.34 per mmHg increase; 95% CI, 1.08–1.67; *P* = 0.009), higher baseline number of glaucoma medications (OR, 1.34; 95% CI, 1.08–1.67; *P* = 0.009), and lower baseline inferior RNFL thickness (OR, 1.28 per 10 μm decrease; 95% CI, 1.03–1.59; *P* = 0.03) were significantly associated with treatment escalation. In multivariable analysis (Table 5), older age (OR, 1.30 per 10 years increase; 95% CI, 1.04–1.63; *P* = 0.02), POAG compared with glaucoma suspect diagnosis (OR, 2.99; 95% CI, 1.59–5.65; *P* < 0.001) and higher baseline IOP (OR, 1.18 per mmHg increase; 95% CI, 1.07–1.30; *P* = 0.01) were significantly associated with treatment escalation.

### Assessment of Potential Visual Field and OCT Progression

This study also examined the outcomes of individual eyes meeting study criteria for worsening visual fields or RNFL thickness to assess if comanagement follow-up accurately identified and managed cases of potentially worsening disease by test measures. Among 180 eyes with at least 3 measurements of RNFL thickness in the study, 14 (7.8%) demonstrated a decline of at least 1 μm/year over the study period. Among 213 eyes with at least 3 visual field measurements of the same testing pattern, 17 (8.0%) had a worsening of mean deviation by at least 1 dB/year. Eyes with worsening testing were either deemed stable by optometrists or rereferred to glaucoma specialists for consideration of treatment escalation. Those eyes in the former group were later evaluated by a glaucoma specialist (Y.H.) during the study review process, who confirmed that potentially worsening testing did not correlate with disease progression requiring escalation of care. For eyes whose treatment was escalated after rereferral, the average time from the optometrist referral to treatment was 27 days for RNFL decline and 20 days for HVF mean deviation decline.

## Discussion

This study investigated the impact of a comanagement system including institutionally trained optometrists and glaucoma fellowship-trained ophthalmologists on the care and stability of selected patients with ocular hypertension or stable glaucoma. Our results demonstrate that, across a 5-year follow-up period, patients managed under this comanagement model exhibited no significant deterioration from baseline in key clinical parameters, including BCVA, mean deviation by HVF, IOP, and OCT RNFL thickness. Furthermore, only a small proportion of patients required escalation of care, and the vast majority returned to the comanagement system after further evaluation by a glaucoma specialist. Our study’s focus on a structured, long-term comanagement system builds on existing literature examining the potential for team-based care in glaucoma management. By assessing the outcomes within a large academic medical center, this study contributes new evidence supporting the sustainability and effectiveness of this model in a cohort of glaucoma suspects or patients with stable glaucoma.

The study’s primary outcome, glaucoma stability, was successfully maintained across both care pathways, with no significant escalation in treatment or disease progression in the majority of patients. This result is consistent with findings from Banes et al^8^ who found that optometrists, when trained within a structured program and overseen by glaucoma specialists, can manage stable patients with glaucoma effectively and safely. Despite some requiring treatment escalation, including laser procedures or surgery, the overall stability across the cohort suggests that in the context of an integrated care delivery system and well-defined referral protocols, optometrists with glaucoma-specific training can medically manage stable glaucoma suspects or early glaucoma patients without detriment to outcomes. Furthermore, patients with findings concerning for worsening disease by HVF or RNFL thickness parameters were appropriately transferred by optometrists―none were incorrectly deemed stable upon later review by a glaucoma specialist. This supports the conclusions of work by Azuara-Blanco et al,^7^ which demonstrated an adequate level of agreement between accredited glaucoma optometrists and ophthalmologists in evaluating glaucoma progression parameters.^5^ We suspect that agreement between optometrists and glaucoma specialists in our study may be even higher due to the intensive glaucoma-specific training provided to participating optometrists. When treatment escalation was required, there were minimal delays in transfer and escalation of care, which did not lead to worsening BCVA or IOP. However, future studies should consider assessing other criteria for rereferral, such as new defects within the central 10° visual field or visual field index (VFI) decline ≥2%/year.

Notably, patients in the alternate care pathway more frequently experienced treatment escalation, such as increased glaucoma medications or laser procedures, compared with those in the transfer pathway, although this difference was not statistically significant. This likely reflects glaucoma specialists’ preferential assignment to the alternate care pathway for patients with greater disease severity or complexity. Indeed, patients in the alternate care pathway were more likely to use IOP-lowering drops and have had a history of laser trabeculoplasty. Patients in the alternate care pathway also required more visits with glaucoma specialists. This suggests that a balance must be struck to appropriately stratify patients by risk. Patients at higher risk of disease progression may benefit from alternate care with direct management guidance from glaucoma specialists. Factors significantly associated with treatment escalation in our study, including older age, a diagnosis of POAG compared with ocular hypertension, and greater baseline IOP, are well described in other literature as risk factors for glaucoma incidence and progression.^13–16^ Further studies should examine the impacts of risk stratification at entry into the comanagement system on efficiency of care utilization without compromising patient outcomes.^17^ Although some comanagement systems in other institutions may follow either a transfer or alternate care structure, it is possible that a dual-pathway system may better balance the need for specialized care with the ability to manage stable disease effectively in a lower-cost setting.

Although institutional differences in comanagement systems may naturally arise, we believe that the success of comanagement relies on clearly defined protocols for identifying the need for treatment escalation and referral, a collaborative working environment, and the active oversight of ophthalmologists or glaucoma specialists. In this study’s system, glaucoma specialists were always available to review optometrists’ questions and equivocal testing results. They also frequently provided oversight with periodic chart review. Without strong glaucoma specialist support and engagement, sustainable comanagement structures are difficult to establish. Furthermore, ophthalmologists should not only guide clinical decision-making in escalated cases but also lead the design, implementation, and ongoing operation of the comanagement framework, establishing standards for care and ensuring that patient outcomes remain the highest priority. Lastly, candidacy for comanagement must be streamlined without compromising patient safety. For example, the LiGHT trial has demonstrated excellent disease stability in patients with mild and moderate glaucoma who have undergone SLT.^18^ Clinical centers could implement a workflow to direct patients matching this archetype toward optometrist-led follow-up.

Although the comanagement system was largely effective in maintaining disease stability, there are opportunities to refine the model. For example, patients with identifiable risk factors for glaucoma progression may benefit from mandatory entry into the alternate care pathway instead of assignment based on a glaucoma specialist’s discretion. Additionally, incorporating home monitoring technology or imaging-only visits might help in identifying progression without increasing the burden of care for optometrists or glaucoma specialists.^19,20^ Lastly, an automated notification system for patients with worsening imaging markers identified by artificial intelligence algorithms may further mitigate missed identification of progression.^21,22^

### Limitations

Several limitations should be considered when interpreting these findings. First, the environment in which this study was conducted―an integrated clinical service within an academic medical center―may not be generalizable to community-based practice. Also, participating optometrists had completed postgraduate clinical training and an intensive glaucoma-specific institutional training program. Therefore, generalizability to other settings or to optometrists without comparable training may be limited. Second, the assignment of patients to transfer versus alternate care reflected real-world discretion rather than rigid criteria, which supports practical implementation but may limit reproducibility across health systems. Based on these findings, our institution is developing standardized entry criteria for future iterations of the program. Third, selection bias may have influenced patient assignment to the alternate or transfer care pathways. Because patients were referred to optometrists at the discretion of their glaucoma specialists, patients in the alternate care pathway were inherently more complex or at higher risk for disease progression. However, we emphasize that the purpose of this study was not to compare outcomes of patients among 2 comanagement groups, but rather to demonstrate stability in clinical parameters of either. Additionally, patients themselves could opt out of inclusion in the comanagement system, introducing another source of selection bias. Next, a glaucoma specialist-only control group was not included. This study focused on longitudinal stability within comanaged patients rather than direct comparison to standard care. However, the absence of a control cohort limits inference on relative efficiency or resource utilization, which will be the focus of future prospective matched studies. A small proportion of eyes included in this analysis underwent cataract surgery during their follow-up period. Because cataract surgery can positively impact visual acuity, visual field performance, and IOP, this may make patients seem stable in these parameters in the setting of glaucomatous disease progression. However, in sensitivity analysis, censoring of eyes that underwent cataract surgery yielded similar results―no significant change in clinical parameters over time. Finally, these findings apply only to glaucoma suspects or patients with stable glaucoma and should not be generalized to advanced or progressing disease.

This study demonstrates that a comanagement system involving optometrists appropriately trained in medical glaucoma management and readily accessible glaucoma specialists can be an effective model for maintaining patient safety and disease stability for patients with glaucoma suspect or stable glaucoma, which comprises the majority of patients seen in a glaucoma practice. This model has the potential to optimize resource utilization and improve patient access, particularly in settings where glaucoma specialist access is limited. By allocating stable glaucoma patients to appropriately trained optometrists, glaucoma specialists can focus on patients with advanced or progressive disease who require timely, specialized care. Future prospective studies with larger, diverse cohorts and randomized control groups are needed to refine patient selection criteria and assess the long-term cost-effectiveness and scalability of comanagement systems in different health care settings and in other subspecialty practices in ophthalmology. In summary, the comanagement model holds promise as a sustainable and efficient approach to glaucoma care, optimizing allocation of limited provider resources and improving patient access to treatment while preserving safety and high standards of clinical care.

## Supplementary Material

1

Supplemental material available at www.ophthalmologyglaucoma.org.

## Figures and Tables

**Figure 1.**
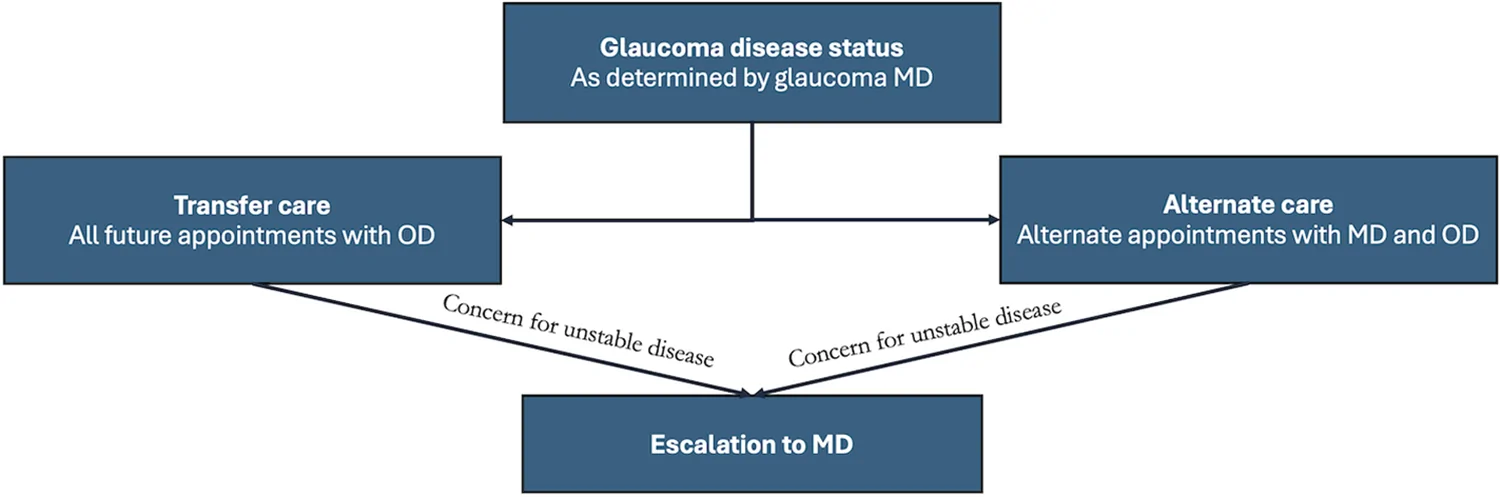
Schematic diagram of the comanagement referral pathway.

**Figure 2.**
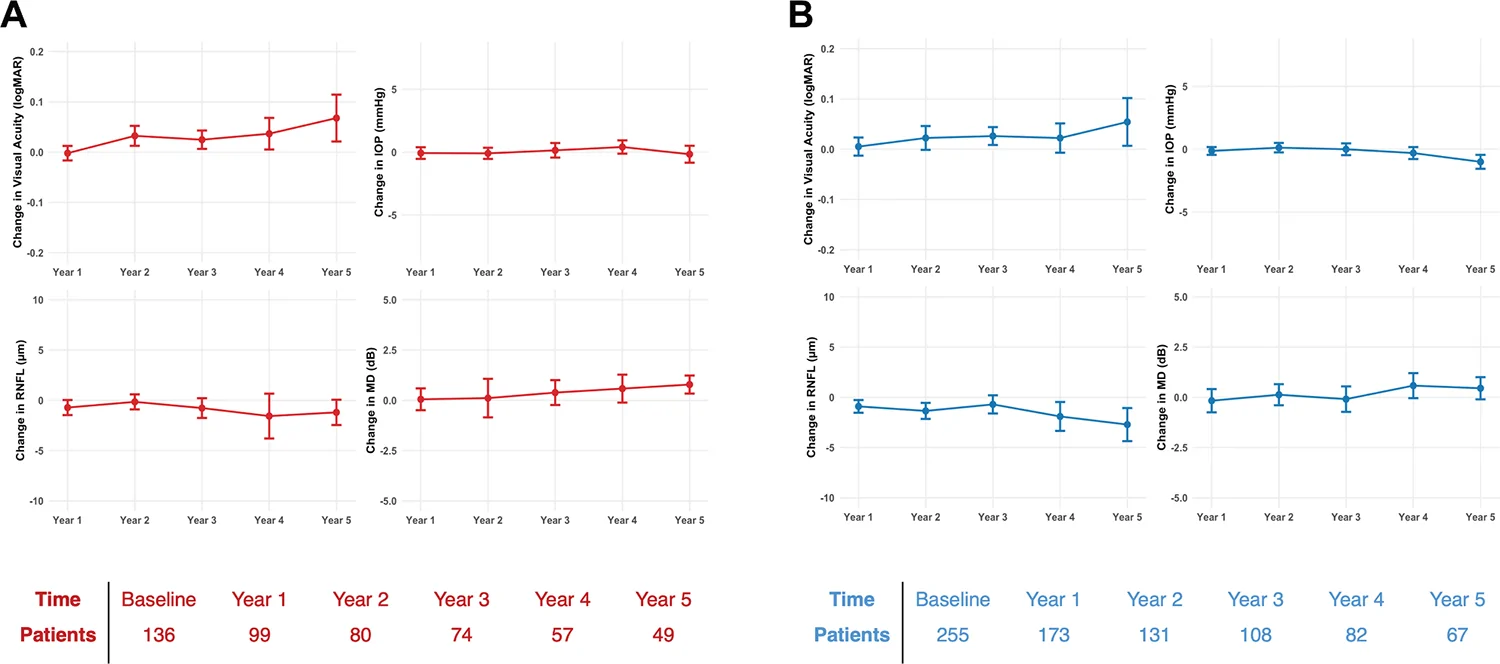
(**A-B**) Mean individual-level change in baseline of visual acuity, intraocular pressure, OCT retinal nerve fiber layer thickness, and Humphrey visual field mean deviation for patients in the alternate care group (**A**) and transfer care group (**B**). Error bars represent 95% confidence intervals (±1.96*standard error).

**Table 1.** Patient-Level Demographic Baseline Characteristics by Comanagement Type

	Total (*n* = 391)	Alternate Care (*n* = 136)	Transfer Care (*n* = 255)	*P* Value*
Age (y) at baseline				0.19
Mean (SD)	71.0 (13.0)	70.1 (12.1)	71.5 (13.5)	
Gender				0.57
Male	185 (47.3%)	67 (49.3%)	118 (46.3%)	
Female	206 (52.7%)	69 (50.7%)	137 (53.7%)	
Self-reported race				0.17
White	126 (32.2%)	47 (34.6%)	79 (31.0%)	
Black	25 (6.4%)	13 (9.6%)	12 (4.7%)	
Asian	143 (36.6%)	46 (33.8%)	97 (38.0%)	
Hispanic	18 (4.6%)	8 (5.9%)	10 (3.9%)	
Unknown	79 (20.2%)	22 (16.2%)	57 (22.4%)	
Year of entry into comanagement system				0.63
2016	9 (2.3%)	5 (3.7%)	4 (1.6%)	
2017	7 (1.8%)	2 (1.5%)	5 (2.0%)	
2018	88 (22.5%)	37 (27.2%)	51 (20.0%)	
2019	108 (27.6%)	36 (26.5%)	72 (28.2%)	
2020	26 (6.6%)	7 (5.1%)	19 (7.5%)	
2021	51 (13.0%)	16 (11.8%)	35 (13.7%)	
2022	63 (16.1%)	20 (14.7%)	43 (16.9%)	
2023	39 (10.0%)	13 (9.6%)	26 (10.2%)	
Years of follow-up data available				
Baseline	391 (100.0%)	136 (100.0%)	255 (100.0%)	
1	272 (69.6%)	99 (72.8%)	173 (67.8%)	
2	211 (54.0%)	80 (58.8%)	131 (51.4%)	
3	182 (46.5%)	74 (54.4%)	108 (42.4%)	
4	139 (35.5%)	57 (41.9%)	82 (32.2%)	
5	116 (29.7%)	49 (36.0%)	67 (26.3%)	

* Comparisons between comanagement group types were performed using unpaired Student *t* tests for continuous measures and chi-squared tests for categorical measures.

**Table 2.** Eye-Level Baseline Clinical Characteristics of Study Participants by Comanagement Type

	Total (*n* = 775 Eyes)	Alternate Care (*n* = 272 Eyes)	Transfer Care (*n* = 503 Eyes)	*P* Value*
Glaucoma diagnosis				<0.001
Glaucoma suspect	397 (51.2%)	100 (36.8%)	297 (59.0%)	
Primary open-angle glaucoma	279 (36.0%)	143 (52.6%)	136 (27.0%)	
PACG/PAC/plateau iris	50 (6.5%)	8 (2.9%)	42 (8.3%)	
Other	49 (6.3%)	21 (7.7%)	28 (5.6%)	
Lens status				0.007
Phakic	350 (45.2%)	151 (55.5%)	199 (39.6%)	
Posterior chamber intraocular lens	423 (54.6%)	120 (44.1%)	303 (60.2%)	
Anterior chamber intraocular lens	2 (0.3%)	1 (0.4%)	1 (0.2%)	
Prior glaucoma laser procedure				0.007
None	607 (78.3%)	202 (74.3%)	405 (80.5%)	
Laser peripheral iridotomy	50 (6.5%)	9 (3.3%)	41 (8.2%)	
Selective laser trabeculoplasty	111 (14.3%)	57 (21.0%)	54 (10.7%)	
Multiple	7 (0.9%)	4 (1.5%)	3 (0.6%)	
History of glaucoma surgery				0.11
None	716 (92.4%)	260 (95.6%)	456 (90.7%)	
Incisional surgery	26 (3.4%)	5 (1.8%)	21 (4.2%)	
Microinvasive glaucoma surgery	25 (3.2%)	4 (1.5%)	21 (4.2%)	
Other	8 (1.0%)	3 (1.1%)	5 (1.0%)	
Best-corrected visual acuity (LogMAR)				0.71
Mean (SD)	0.14 (0.36)	0.13 (0.32)	0.14 (0.33)	
Intraocular pressure (mmHg)				0.65
Mean (SD)	15.5 (3.5)	15.6 (4.1)	15.5 (3.2)	
Number of glaucoma medications				0.003
Mean (SD)	0.7 (1.0)	1.0 (1.0)	0.6 (1.0)	
Mean deviation on HVF (dB)	−2.9 (4.5)	−2.9 (3.9)	−2.9 (4.8)	0.87
Mean (SD)				
Mean deviation stratification				
≥ −6 dB	356 (84.0%)	132 (85.7%)	224 (83.0%)	0.72
< −6 dB and ≥ −12 dB	42 (9.9%)	13 (8.4%)	29 (10.7%)	
< −12 dB	26 (6.1%)	9 (5.8%)	17 (6.3%)	
Pattern standard deviation on HVF (dB)				0.64
Mean (SD)	3.4 (3.0)	3.5 (3.2)	3.3 (2.8)	
Average RNFL on OCT (μm)				0.65
Mean (SD)	86.2 (12.7)	85.8 (11.6)	86.5 (13.2)	

HVF = Humphrey visual field; PAC = primary angle closure; PACG = primary angle closure glaucoma; RNFL = retinal nerve fiber layer.

* From logistic regression model with intereye correlation adjusted by using generalized estimating equations.

**Table 3.** Trends in Care by Comanagement Group

	Alternate Care	Transfer Care	*P* Value
Overall trend of care	*n* = 136 patients	*n* = 255 patients	
Remained transfer pathway	-	214 (83.9%)	
Remained alternate pathway	87 (64.0%)	-	
Converted from alternate to transfer pathway	34 (25.0%)	-	
Rereferral to MD and eventually transferred back to OD	13 (9.6%)	30 (11.8%)	
Rereferral to MD and remained under MD care	2 (1.5%)	11 (4.3%)	
Treatment escalation during the study period	*n* = 272 eyes	*n* = 503 eyes	
Increase in glaucoma medication regimen	36 (13.2%)	45 (8.9%)	0.17
Glaucoma-related laser procedure	14 (5.1%)	20 (4.0%)	0.54
Glaucoma surgery	12 (4.4%)	5 (1.0%)	0.02

MD = glaucoma specialist; OD = optometrist.

**Table 5.** Multivariable Analysis for Factors Associated with Treatment Escalation

Factors	Category or Increment	Adjusted OR (95% CI)	*P* Value
Age	Every 10-yr increase	1.30 (1.04–1.63)	0.02
Glaucoma diagnosis	Glaucoma suspect	1.00	
	Primary open-angle glaucoma	2.99 (1.59–5.65)	<0.001
	PAC/PACG/plateau iris	0.99 (0.20–4.93)	0.99
	Other	2.59 (0.89–7.52)	0.08
Baseline IOP	Every 1 mmHg increase	1.18 (1.07–1.30)	0.01

Factors with *P* value ≤ 0.20 from univariable analysis were included in the initial multivariable model, and only factors with *P* value < 0.05 in the final multivariable model were reported in this table.

CI = confidence interval; IOP = intraocular pressure; OR = odds ratio; PAC = primary angle closure; PACG = primary angle closure glaucoma.

## Data Availability

The research data is confidential, therefore we cannot provide the data for open access. This study were presented at the 2025 ARVO Meeting; May 4–8, 2025; Salt Lake City, Utah, 2025 AGS Meeting; February 26-March 2, 2025, Washington D.C.

## Abbreviations and Acronyms:

BCVA: best-corrected visual acuity
CI: confidence interval
HIPAA: Health Insurance Portability and Accountability Act
HVF: Humphrey visual field
IOP: intraocular pressure
IQR: interquartile range
IRB: Institutional Review Board
MMG: mixed mechanism glaucoma
OR: odds ratio
POAG: primary open-angle glaucoma
RNFL: retinal nerve fiber layer
SD: standard deviation
SLT: selective laser trabeculoplasty
UCSF: University of California San Francisco
