# Supplementary material for: Ophthalmologist and Optometrist Comanagement Model for Glaucoma: Outcomes from a Single Tertiary Care Health Center
Source: Ophthalmol Glaucoma. Author manuscript; Available in PMC 2026 Oct 23. (PMC13694102; doi:10.1016/j.ogla.2026.02.002)
Supplement: Supplementary file 1 [file NIHMS2211361-supplement-1.pdf]

Supplemental Table 4. Univariable analysis for factors associated with treatment escalation

Factors	Category or increment	Adjusted OR (95% CI)	p value
**Age**	Every 10-year increase	1.29 (1.07, 1.56)	0.008
**Gender**	Male	1.00	
	Female	1.09 (0.65, 1.84)	0.74
**Co-management type**	transfer care	1.00	
	alternating care	1.87 (1.11, 3.16)	0.02
**Glaucoma diagnosis**	Glaucoma suspect	1.00	
	Primary open angle glaucoma	2.99 (1.59, 5.65)	<0.001
	PAC/PACG/plateau iris	0.99 (0.20, 4.93)	0.99
	Other	2.59 (0.89, 7.52)	0.08
**Other ocular disease**	No	1.00	
	Yes	0.92 (0.53, 1.58)	0.76
**Phakic status**	Phakic	1.00	
	Pseudophakic	1.08 (0.64, 1.82)	0.76
**History of laser procedure**	None	1.00	
	LPI	0.58 (0.20, 1.70)	0.32
	SLT	2.48 (1.36, 4.50)	0.003
	Multiple	2.67 (0.29, 25.06)	0.39
**Baseline Best VA (logMAR)**	Every 0.1 logMAR increase	1.01 (0.96, 1.07)	0.6
**Baseline IOP**	Every 1 mmHg increase	1.18 (1.07, 1.30)	0.01
**Baseline # of glaucoma medications**	Every 1-unit increase	1.34 (1.08, 1.67)	0.009
**Baseline MD**	Every 1-unit decrease	0.998 (0.925, 1.077)	0.96
**Baseline PSD**	Every 1-unit increase	1.01 (0.92, 1.10)	0.85
**Baseline VFI**	Every 10-unit increase	0.98 (0.74, 1.29)	0.87
**Baseline Average RNFL**	Every 10-unit increase	0.79 (0.60, 1.03)	0.08
**Baseline Superior RNFL**	Every 10-unit increase	0.81 (0.63, 1.06)	0.13
**Baseline Inferior RNFL**	Every 10-unit increase	0.78 (0.63, 0.97)	0.03
**Baseline Average GCC**	Every 10-unit increase	0.95 (0.69, 1.30)	0.75

Factors with p value ≤ 0.20 from univariable analysis were included in the initial multivariable model, and only factors with p value < 0.05 in the final multivariable model were reported in this table.

OR – odds ratio; CI – confidence interval; IOP – intraocular pressure; PAC – primary angle closure; PACG – primary angle closure glaucoma; LPI – laser peripheral iridotomy; SLT – selective laser trabeculoplasty; VA – visual acuity; IOP – intraocular pressure; MD – mean deviation; PSD – pattern standard deviation; VFI – visual field index; RNFL – retinal nerve fiber layer; GCC – ganglion cell complex
